# P33 Using the Australian National Antimicrobial Prescribing Survey (NAPS) portal to undertake an annual point prevalence survey (PPS)

**DOI:** 10.1093/jacamr/dlad066.037

**Published:** 2023-06-14

**Authors:** Bee Yean Ng, Teig Parsons, Bernadette O’Riordan, Gemma Pill, Louise Dunsmure, Nicola Jones

**Affiliations:** Antimicrobial Stewardship Team; Antimicrobial Stewardship Team; Antimicrobial Stewardship Team; Antimicrobial Stewardship Team; Antimicrobial Stewardship Team; Antimicrobial Stewardship Team; Infectious Diseases Department, Oxford University Hospitals NHS Foundation Trust, UK

## Abstract

**Background:**

Annual point prevalence surveys (PPS) are cornerstones in antimicrobial stewardship (AMS). The National Antimicrobial Prescribing Survey (NAPS) is a web-based qualitative auditing platform that provides a standardized and validated tool to assist hospitals in assessing the appropriateness of antimicrobial prescribing.

**Objectives:**

To evaluate the usefulness of NAPS in a UK tertiary hospital setting.

**Methods:**

Permission to use the platform was given by NAPS on a pilot basis. NAPS e-learning was completed by the AMS team. All patients on antimicrobials on a single day were extracted using the Cerner electronic prescribing system. Indication, appropriateness and compliance to guidelines were reviewed, anonymized and uploaded by the trained individuals to NAPS. Ethical approval was not required.

**Results:**

695 patients were audited. 35% (*n* = 241) of the patients were prescribed antimicrobials. The total number of antimicrobial prescriptions was 326, and 46% (*n* = 190) of antimicrobial prescriptions were under general medicine and geratology. The most common indications for antimicrobials were respiratory 27% (*n* = 87) followed by intra-abdominal infection 11% (*n* = 37), sepsis and bacteraemia 11% (*n* = 37), urinary tract infection 10% (*n* = 34) and skin and soft tissue infection 8% (*n* = 25). The most prescribed agent was co-amoxiclav 34% (*n* = 111). Compliance with guidelines was achieved in 84% (*n* = 273) of prescriptions, and 72% (*n* = 236) were deemed appropriate. Inappropriate prescriptions were more prevalent for co-amoxiclav (38%) and ceftriaxone (43%) compared with amoxicillin (6%) and doxycycline (8%) (Figure 1). The most common reasons for inappropriate prescription were incorrect (prolonged) duration (44%) and spectrum too broad (28%).

**Conclusions:**

Using NAPS, we identified that general medicine and geratology are the major users of antimicrobials, and pneumonia is the leading indication for prescribing. Co-amoxiclav is the main antibiotic prescribed, and appropriateness of this and other antibiotics, such as ceftriaxone, are below the 90% target. Consequently, targeted AMS efforts can be designed. PPS results are feedback to the respective teams and have been used in teaching sessions to encourage teams to take ownership of their data and influence prescribing patterns. We have found NAPS to be user friendly and reliable. Reports are produced immediately on completion of the survey to allow timely feedback. We would recommend NAPS as a useful tool in performing PPS and as a potential way to benchmark NHS trusts.

**Figure 1.**
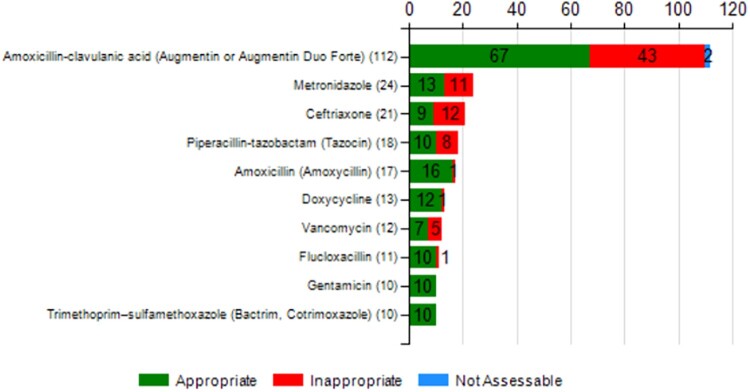
Appropriateness of prescriptions.

